# FRET-Based Identification of mRNAs Undergoing Translation

**DOI:** 10.1371/journal.pone.0038344

**Published:** 2012-05-31

**Authors:** Benjamin Stevens, Chunlai Chen, Ian Farrell, Haibo Zhang, Jaskiran Kaur, Steven L. Broitman, Zeev Smilansky, Barry S. Cooperman, Yale E. Goldman

**Affiliations:** 1 Pennsylvania Muscle Institute, School of Medicine, University of Pennsylvania, Philadelphia, Pennsylvania, United States of America; 2 Anima Cell Metrology, Inc., Bernardsville, New Jersey, United States of America; 3 Department of Chemistry, University of Pennsylvania, Philadelphia, Pennsylvania, United States of America; 4 Department of Biology, West Chester University of Pennsylvania, West Chester, Pennsylvania, United States of America; The John Curtin School of Medical Research, Australia

## Abstract

We present proof-of-concept *in vitro* results demonstrating the feasibility of using single molecule fluorescence resonance energy transfer (smFRET) measurements to distinguish, in real time, between individual ribosomes programmed with several different, short mRNAs. For these measurements we use either the FRET signal generated between two tRNAs labeled with different fluorophores bound simultaneously in adjacent sites to the ribosome (tRNA-tRNA FRET) or the FRET signal generated between a labeled tRNA bound to the ribosome and a fluorescent derivative of ribosomal protein L1 (L1-tRNA FRET). With either technique, criteria were developed to identify the mRNAs, taking into account the relative activity of the mRNAs. These criteria enabled identification of the mRNA being translated by a given ribosome to within 95% confidence intervals based on the number of identified FRET traces. To upgrade the approach for natural mRNAs or more complex mixtures, the stoichiometry of labeling should be enhanced and photobleaching reduced. The potential for porting these methods into living cells is discussed.

## Introduction

The final step in protein expression in cells is mRNA-programmed synthesis of proteins by the ribosome. As regulation of protein expression is a major factor controlling cellular development and responses to environmental cues, methods for measuring protein expression levels in cells have been extensively pursued. Well-established tools for identifying and quantifying proteins in cell extracts include 1D- and 2D-gels, DNA microarrays and mass spectrometry, often coupled with the use of radioactive or stable-isotope-labeled amino acids [1]. A recent approach, ribosome-profiling, targets the translation machinery and identifies mRNAs that are undergoing translation at a given point in time [2]. In addition, fluorescence methods for analyzing protein synthesis within intact cells are also available, either via fusion of the target protein with fluorescent reporter proteins [3], [4], [5] or peptides that can be labeled specifically with smaller, bright organic dyes [6], [7], [8], [9]. These methods, though quite powerful, have significant limitations. The non-fluorescent approaches only work on cell extracts, often requiring tedious preparation techniques that would be very demanding to use for obtaining kinetic data. The fluorescence methods report the amount of protein accumulated in the cell, rather than the rate of synthesis, and after some delay, since labeling is generally slow compared to the rate of synthesis. In addition, mutations required for fluorescent label incorporation, or the label itself, may, in some cases, affect both the accumulation and distribution of the target protein. Recently tRNAs, labeled with several different fluorophores, were detected binding to single ribosomes, using highly specialized instrumentation [10], suggesting that the sequence of the individual peptide being synthesized could be deduced from these signals.

Here we set out to study the feasibility of using single molecule fluorescence resonance energy transfer (smFRET) measurements to distinguish, in real time, between ribosomes programmed with several different, short mRNAs. We used two distinct FRET approaches, a) the FRET signal generated between two tRNAs labeled with different fluorophores bound simultaneously to the ribosome (tRNA-tRNA FRET) [11], [12], [13] and b) the FRET signal generated between a labeled tRNA bound to the ribosome and a fluorescent derivative of ribosomal protein L1 (L1-tRNA FRET) [14], [15]. Suitably extended, either approach should enable *in vitro* identification of mRNAs undergoing translation in complex mixtures (e.g., those derived from cellular or sub-cellular extracts). In addition, the L1-tRNA approach has the potential to enable monitoring the rates of synthesis of individual proteins in live cells in real time.

## Results

### Event identification by tRNA-tRNA and L1-tRNA FRET measurements

Single ribosomes were tethered onto a microscope coverslip through 3′-biotinylated (mRNAs -1, -2, -5, and -6) or 5′-biotinylated (mRNAs -3 and -4) mRNAs as described in Methods. Fluorescence signals from labeled Phe-tRNA^Phe^s, Val-tRNA^Val^s and labeled ribosomal protein L1 were observed by total internal reflection fluorescence (TIRF) microscopy when the labeled tRNAs were bound to the surface immobilized ribosomes. The tRNAs were labeled at dihydrouridine (DHU) residues falling within the D-loop. We have shown that such labeled tRNAs containing ∼1 dye/tRNA retain functionality in protein synthesis assays [12], [16], [17]. The ribosomes were programmed with model mRNAs (Table 1) that encode sequences with adjacent Phe (F) and Val (V) codons, as well as with Tyr (Y), Arg (R), Glu (E) and Met (M) codons. The mRNAs could be distinguished from one another by the order of appearance of the corresponding fluorescent signals from FV or VF pairs, along with FRET between pairs of adjacent tRNAs or between L1 and tRNAs.

**Table 1:** mRNAs used in this study.

mRNA	Coded peptide sequences	mRNA sequence
1	M RFV (YFV)_6_ RFV (YFV)_6_	GGG AAU UCG AAA UAG AAG UCU UCU UUU UGG A AAA AUU UAA AAG UUA AUA AGG AUA CAU ACU ***AUG*** CGU UUU GUG UAU UUU GUG UAU UUU GUG UAU UUU GUG UAU UUU GUG UAU UUU GUG UAU UUU GUG CGU UUU GUG UAU UUU GUG UAU UUU GUG UAU UUU GUG UAU UUU GUG UAU UUU GUG UAA CGC GUC UGC AGG CAU GCA AGC UAA AAA AAA AAA AAA AAA AAA AAA AAA GCU
2	M RVF (YVF)_6_ RVF (YVF)_6_	GGA AAA AUU UAA AAG UUA AUA AGG AUA CAU ACU ***AUG*** CGU GUG UUC UAU GUG UUC UAU GUG UUC UAU GUG UUC UAU GUG UUC UAU GUG UUC UAU GUG UUC CGU GUG UUC UAU GUG UUC UAU GUG UUC UAU GUG UUC UAU GUG UUC UAU GUG UUC UAU GUG UUC UAA CGC GUC UGC AGG CAU GCA AGC UAA AAA AAA AAA AAA AAA AAA AAA AGG ATC CCT AGC ATA ACC CCT TGG GGC CTC TAA ACG GGT CTT GAG GGG TTT TTT GA
3	MRFVRFVRF	GGG AAU UCA AAA AUU UAA AAG UUA AUA AGG AUA CAU ACU ***AUG*** CGU UUC GUG CGU UUC GUG CGU UUC
4	MFRVFRVFR	GGG AAU UCA AAA AUU UAA AAG UUA AUA AGG AUA CAU ACU ***AUG*** UUC CGU GUG UUC CGU GUG UUC CGU
5	M (VE)_6_ (RFV)_2_ RFK (RFV)_2_ RFM	AAU UCA AAA AUU UAA AAG UUA AUA AGG AUA CAU ACU ***AUG*** GUU GAA GUU GAA GUU GAA GUU GAA GUU GAA GUU GAA CGU UUU GUU CGU UUU GUU CGU UUU AAA CGU UUU GUU CGU UUU GUU CGU UUU AUG UUC UUC UUC UUC UUU UUU UUU GUCUUC CUG CAG UUU UUU UUU UUU UUU UUU UUU UUU UUU A
6	M RFV YVF (YFVYVF)_3_ RFV YVF (YFVYVF)_3_	GGA AAA AUU UAA AAG UUA AUA AGG AUA CAU ACU ***AUG*** CGU UUU GUG UAU GUG UUC UAU UUU GUG UAU GUG UUC UAU UUU GUG UAU GUG UUC UAU UUU GUG UAU GUG UUC CGU UUU GUG UAU GUG UUC UAU UUU GUG UAU GUG UUC UAU UUU GUG UAU GUG UUC UAU UUU GUG UAU GUG UUC UAA CGC GUC UGC AGG CAU GCA AGC UAA AAA AAA AAA AAA AAA AAA AAA AGG ATC CCT AGC ATA ACC CCT TGG GGC CTC TAA ACG GGT CTT GAG GGG TTT TTT GA

The bold sequence for each mRNA is the translation start site, initiator fMet. Sequences are listed from 5′ to 3′ ends. The biotin tag for tethering to the microscope slide surface was at the 3′ end for mRNAs -1, -2, -5, and -6, and at the 5′ end for mRNAs -3 and -4.

**Figure 1:**
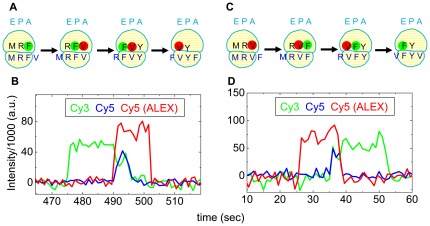
Single FV and VF events detected by FRET between Cy3-F and Cy5-V during translation of mRNA-1 (FV: A, B) and mRNA-2 (VF: C, D). In the cartoons (A, C), fluorescent labels are shown as filled colored circles: Cy3 is green, Cy5 is red. Black and blue letters represent the tRNAs and codon triplets, respectively, for a given amino acid, using the standard single-letter abbreviations. For ease of presentation, all three tRNA sites are shown to be occupied during the entire event, but this need not be the case [13]. The excitation wavelength was alternated (ALEX) between 532 nm and 640 nm every other image. The traces in (B, D) show Cy3 fluorescence (green trace, 585 nm detection) and sensitized emission of Cy5 (FRET, blue trace, 680 nm detection), both under 532 nm excitation, and Cy5 fluorescence (red ALEX trace, 680 nm detection) under direct 640 nm excitation.

The cartoons in Fig. 1A,C illustrate several steps of elongation for ribosomes programmed with mRNA-1 (….YFVYFVYFV…., Table 1) or mRNA-2 (…YVFYVFYVF …, Table 1), respectively. Figs. 1B,D show two sections of recordings of tRNA-tRNA fluorescence traces with Cy3 labeled Phe-tRNA^Phe^ (denoted Cy3-F) and Cy5 labeled Val-tRNA^Val^ (Cy5-V) during elongation by ribosomes programmed with mRNA-1 and mRNA-2, respectively. In Fig. 1B initial Cy3-F binding is indicated by an increase in the green trace (direct Cy3 fluorescence under 532 nm excitation). Following accommodation and translocation, the subsequent binding of Cy5-V is indicated by three simultaneous fluorescence changes: the red trace (direct Cy5 fluorescence under 640 nm illumination) and the blue trace (sensitized emission (FRET) of Cy5 under 532 nm illumination) increase, whereas the Cy3 fluorescence (green) decreases as the Cy3-F donates energy to Cy5-V. Next, FRET and Cy3 fluorescence decrease simultaneously to the baseline when the deacylated Cy3-F dissociates after another translocation step. Finally, Cy5 fluorescence drops when the deacylated Cy5-F dissociates. Dissociation of deacylated tRNA from the E-site likely precedes binding of the next ternary complex under these conditions [13]. The trace in Fig. 1D demonstrates, as expected, the inverse sequence of fluorescence changes. The order and synchronization of appearance of the three fluorescent signals unambiguously distinguish between Cy3-Cy5 (FV) and Cy5-Cy3 (VF) events during elongation and hence between mRNA-1 and mRNA-2.

**Figure 2 pone-0038344-g002:**
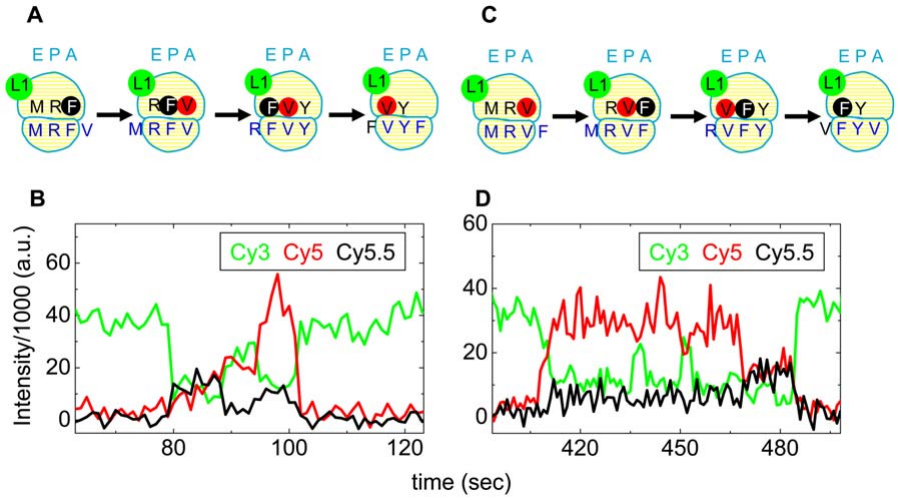
Single FV and VF events detected by FRET between L1^Cy3^ and either Cy5.5-F or Cy5-V during translation of mRNA-1 (FV: A, B) and mRNA-2 (VF: C, D). The cartoons (A, C) are presented as in Fig. 1, with the addition that Cy5.5 is black. The traces (B, D) show Cy3 fluorescence (green) and sensitized emission of Cy5 (FRET, red) and Cy5.5 (FRET, black), all under 532 nm excitation. The emission filter wavelengths are listed in Methods. In B, The proximity of Cy5.5 to Cy3 causes an increase in intensity in the Cy5.5 sensitized emission channel, with some cross-talk into the Cy5 channel, followed by release of the Cy5.5-F and closer approach of the Cy5 dye to Cy3, followed by release of Cy5. In D, the order is reversed.

Similar cartoon and event recording panels in Fig. 2 illustrate donor fluorescence from Cy3-labeled L1 (L1^Cy3^, green) and sensitized emission from Cy5-V (red) and Cy5.5-labeled Phe-tRNA^Phe^ (Cy5.5-F, black) with panels A, B and C, D corresponding to ribosomes programmed with mRNA-1 or mRNA-2, respectively. Although Cy5 and Cy5.5 overlap spectrally, they can be distinguished by the different intensities in the respective detector channels. Similarly to the tRNA-tRNA fluorescent traces, the L1-tRNA FRET traces enable distinction between VF and FV events and thus between mRNA-1 and mRNA-2.

**Figure 3:**
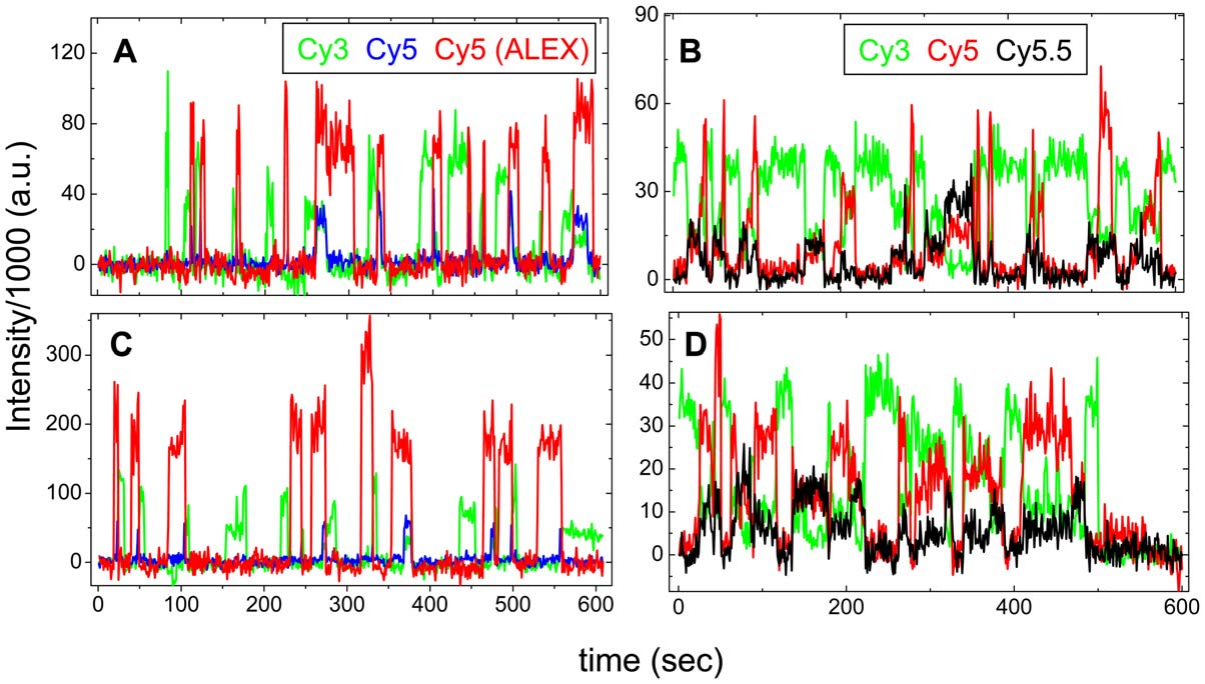
Multiple FV and VF events during translation of mRNA-1 and mRNA-2. (A) and (B), translation of mRNA-1, as detected by tRNA-tRNA FRET and by L1-tRNA FRET, respectively. (C) and (D), translation of mRNA-2, as detected by tRNA-tRNA FRET and by L1-tRNA FRET, respectively. Color coding: (A) and (C), as in Fig. 1; (B) and (D), as in Fig. 2.

The individual events in Figs. 1 and 2 were taken from longer recordings showing repeated tRNA-tRNA (Fig. 3A, C) and L1-tRNA (Fig. 3B, D) events during translation of mRNA-1 and mRNA-2. The repeating pattern of FV or VF events in these traces, separated by periods of no tRNA-tRNA fluorescence or L1-tRNA FRET during decoding of unlabeled tRNAs, distinguishes them unambiguously. We categorize repeating FV and VF events as ‘criterion events’ because they indicate whether a ribosome is translating mRNA-1 or mRNA-2.

In experiments with mRNA-1 or mRNA-2 small percentages of events corresponding to the opposite order of FV or VF expected from that mRNA were detected. For mRNA-1 the percentage of mischaracterized traces were 12% (tRNA-tRNA) and 3% (L1-tRNA) and for mRNA-2, 5% (tRNA-tRNA) and 0% (L1-tRNA), respectively. These errors of mRNA identification are unlikely to stem from erroneous detection of the fluorophores, because all the ordered events shown in Figs. 1B, 1D, 2B and 2D were clearly detected in the signals. In most cases, mischaracterization rates for the L1-tRNA approach were lower than that for the tRNA-tRNA approach (Table 2). Misidentification, at least for the tRNA-tRNA approach, could be caused by non-specific, simultaneous binding of two labeled tRNAs on the surface of the microscope slide, for instance at surface defects and inactive ribosome complexes. In the L1-tRNA approach, the contribution from non-specific binding is expected to be much less, because the correct FRET signals can only be generated when labeled tRNAs translocate through active ribosomes. Another possible cause of misidentification is contamination of the commercial Val-tRNA preparation with Phe-tRNA and *vice versa*. Contaminating tRNAs would become labeled in the preparative procedure, but the proportion of charged, labeled contaminating tRNAs would be very low.

**Table 2:** Parameter values for Eq. 1 (Fig. 4).

Panel in Fig. 4	X^a^	Y^a^	R^b^		T_n100_		T_n0_	
			tRNA-tRNA	L1-tRNA	tRNA-tRNA	L1-tRNA	tRNA-tRNA	L1-tRNA
A	1	2	0.39	1.84	0.88	0.97	0.05	0
B	3	5	12.0	7.31	0.84	0.96	0.06	0
C	3	4	4.8	3.72	0.97	0.97	0.17	0.029

a X, Y refer to specific mRNAs (see Table 1).

b R is the ratio of the apparent synthetic activity of mRNA-X relative to mRNA-Y in the mixture. Deviations of R from 1.0 may reflect differences in the efficiencies of initiation complex formation and of polypeptide elongation, the higher probability of detecting longer mRNAs vs. shorter mRNAs, and other factors not yet identified.

#### Criterion events for other mRNAs

Fluorescence traces recorded for ribosomes programmed with mRNAs-3–5, similar to those shown in Figs. 1–3, are shown in Fig. S1 (tRNA-tRNA: Cy3-F, Cy5-V) and Fig. S2 (L1-tRNA: L1^Cy3^, Cy5.5-F, Cy5-V). The repeating FV and VF patterns found for mRNA-3 and -4 are similar to those found for mRNA-1 and -2, respectively, except they were produced at lower frequency per trace due to the shorter lengths of mRNAs-3 and -4. Several single V binding events were found before the first FV event with mRNA-5. These observed ‘criterion events’ are consistent with the mRNA sequences.

The validity of assigning a specific mRNA to a recording containing a given criterion event depends on codon-dependent binding of fluorescent tRNAs to the ribosome via the A-site. We found that buffers containing polyamines facilitated spurious binding events (Fig. S3), unrelated to polypeptide elongation, that are most likely due, at least in part, to deacylated tRNA binding to the E-site [18], [19]. In contrast, a buffer lacking polyamine and containing relatively high Mg^2+^ concentration (15 mM) dramatically decreases such spurious binding while supporting good ribosome activity (Fig. S3), so this buffer was used in all the further experiments presented herein.

### Using criterion events to identify ribosomes translating a given mRNA from a mixture of two different mRNAs

**Figure 4:**
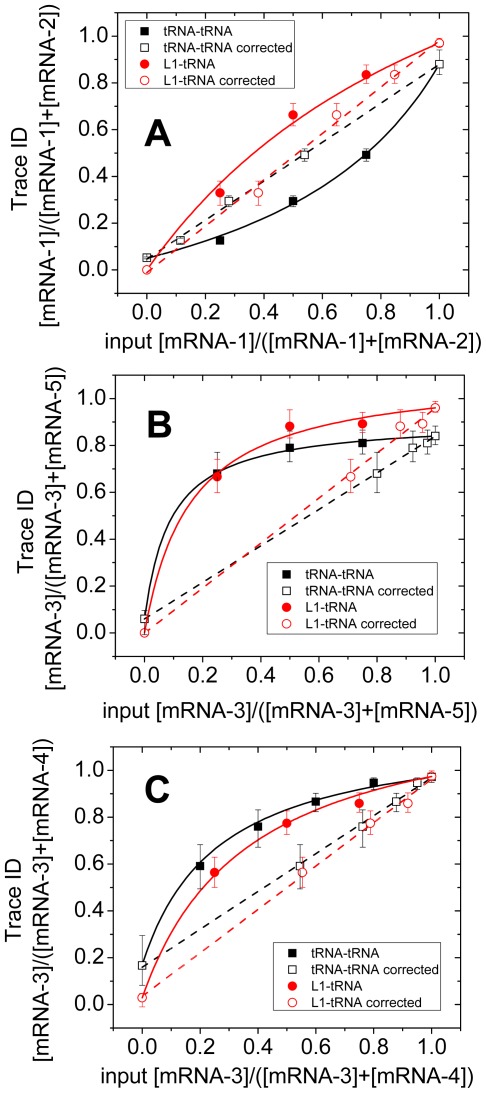
Analysis of mixtures of two input mRNAs. In all three panels, the proportion of one identified translated mRNA (Trace ID) is plotted (filled symbols) against the proportion of that mRNA in the reaction mixture (input proportion). The solid line through these points is fitted from Eq. 1 in the text, adjusting the ratio (R) of efficiencies of translation of the two mRNAs. The open symbols show corresponding corrected effective input proportions from Eq. 2 using the values for R from the fitted Eq. 1. tRNA-tRNA FRET (squares); L1-tRNA FRET (circles). The dashed straight lines are fitted to the corrected input proportions. mRNA mixtures: (A) 1 and 2; (B) 3 and 5; (C) 3 and 4. Uncertainties are 95% confidence intervals [37].

The results presented in Figs. 1–3, S1 and S2 demonstrate differences expected in the fluorescence signals observed during translation of mRNAs 1–5. Fig. 4 demonstrates that criterion events observed within a given trace can be used to identify the mRNA being translated by a ribosome from within three different mixtures of initiation complexes (70SICs), each formed from two different mRNAs and then mixed together. The total concentration of 70SICs was held constant, while the proportions of the 70SICs within the mixture, derived from different mRNAs, were varied. The proportions of traces identified as arising from one or the other of the 70SICs was determined using criterion events. For the mixture containing mRNA-1 and -2, which 70SIC was being elongated was identified by whether there are more FV than VF events within the trace (mRNA-1) or vice-versa (mRNA-2) as measured by either tRNA-tRNA or L1-tRNA FRET (Fig. 4). The various 70SICs showed different translational activities when used alone, or in mixtures, because of different elongation rates that likely depend on some combination of codon sequence and the intrinsic activities of the labeled charged tRNAs.

Assuming that differences in detection of each mRNA from that expected from the proportions of 70SICs are due entirely this differential activity and that there is no interference between the 70SICs in the mixtures leads to the following equation for event detection:
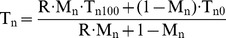
(1)where n refers to the mRNA number (1, 2, …5; Table 1), T_n_ = the proportion of traces identified as arising from mRNA-n, R = the elongation rate of mRNA-n relative to the other template in the mixture, M_n_ = the input mRNA fraction, T_n100_ = proportion of traces correctly identified as mRNA-n in the 100% mRNA-n case, and T_n0_ = proportion of traces wrongly identified as mRNA-n in the 0 mRNA-n case. The small deviations of T_n100_ and T_n0_ from 1.0 and 0.0, respectively, represent the intrinsic errors mentioned above in identifying traces via criterion events.

Detected events describe curved relationships with respect to the proportions of the 70SICs (Fig. 4). These relationships are well fit by Eq. 1 when R is an adjustable parameter (Table 2), clearly indicating that the deviations from linearity in Fig. 4 are due to variations in translational activities among the 70SICs and that the results obtained are compatible with the assumptions listed above.

The input mRNA-n fraction M_n_ can be transformed into a corrected input fraction M_n_′ that takes into account the relative elongation rates via Eq. 2
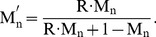
(2)


The validity of this procedure is demonstrated by the close fit (within 95% confidence intervals) of the identified mRNAs to the straight lines obtained for plots of T_n_ vs. M_n_′, Fig. 4A–C, indicating proportions of translation of two mRNAs by ribosomes programmed with the three mRNA mixtures.

In analyses of traces recorded from the other mixtures of mRNAs, the criterion events for classifying traces were: for mRNA-5 vs. mRNA-3 (Fig. 4B), the occurrence of isolated V binding events preceding FV events and for mRNA-3 vs. mRNA-4 (Fig. 4C), the occurrence of more FV than VF events as for mRNA-1 vs. mRNA-2 (Fig. 4A). In each case, the observed plots of T_n_ against M_n_ were well fit to Eq. 1, and T_n_ plotted vs. M_n_′ approximated a straight line within the 95% confidence limits for most of the trace identifications. Parameter values for the plots in Fig. 4 are collected in Table 2. T_n100_ and T_n0_ values were within a few percent of 1.0 and 0.0, respectively except in one case discussed below. Relative activities of the mRNAs ranged from 0.4–12-fold for unknown reasons. The straight lines in Fig. 4 indicate that identifications of the mRNAs translated by the individual ribosomes follow those expected from these relative activities.

A 6^th^ mRNA containing alternating FV and VF pairs separated by codons for unlabeled tRNAs was also compared in tRNA-tRNA experiments with mRNA-2, which contains only VF pairs of labeled-tRNA codon pairs (Fig. S4A). However, distinguishing mRNA-6 from mRNA-2, using the alternating FV and VF events as the criterion, led to a high rate of misidentification (∼20%, Fig. S4B), because identification requires observation of at least four labeled tRNAs binding to the ribosome and is thus more sensitive to substoichiometric tRNA labeling than the other mRNAs.

## Discussion

Here we successfully tested the proposition, albeit with a quite simple RNA library, that specific polypeptide sequences can be distinguished from one another, while they are being synthesized in real-time, by capturing and analyzing tRNA-specific fluorescence and FRET signals from individual ribosomes. The FRET signals were generated using two labeling approaches, giving rise to either tRNA-tRNA FRET pairs or to L1-tRNA FRET pairs. Our results highlight the main challenges that must be met to make these methods applicable to monitoring protein synthesis in general. The use of FRET in the tRNA-tRNA and L1-tRNA approaches is an important distinction between this work and that of a recent report, Uemura et al. [10], who demonstrated how single molecule fluorescence intensity measurements alone could be used to identify an mRNA undergoing translation. In that work, zero mode waveguides and a specialized illumination system were used, limiting the detection to the sample surface, whereas the FRET method could be applied to thicker samples, such as biological cells. Sensitized acceptor emission in FRET has high signal-to-background ratio and some of us have taken advantage of this feature in a report of tRNA-tRNA FRET measurements of protein synthesis rate in live cells [20], albeit not at the single molecule level. A second distinction is that Uemura et al. did not analyze mRNA mixtures, as is demonstrated here.

For our current *in vitro* TIRF experiments, the tRNA-tRNA approach exhibits two major advantages compared to the L1-tRNA approach. First, photobleaching is more limited since any individual labeled tRNA is only briefly illuminated by the excitation laser beams while it is bound to the ribosome, allowing for prolonged observation of the translating ribosome and potentially long reads of mRNA sequence. In contrast, the fluorophore attached to ribosome is continually illuminated in the L1-tRNA approach, limiting the number of cycles than can be observed prior to photobleaching. Labeling the ribosomes with longer lasting fluorescent probes such as quantum dots [21] or weakly bound, exchangeable, organic fluorophores [22] would mitigate this difference. Second, in L1-tRNA experiments, all labeled ribosomes are observed, both active and inactive, whereas tRNA-tRNA FRET allows pre-selection of only active ribosomes for observation.

On the other hand, the fraction of observed FRET events to the total expected number is higher for L1-tRNA than for tRNA-tRNA, because it is affected to a lesser extent by incomplete labeling of the tRNAs and competition between labeled and unlabeled tRNAs for the A-site. The combined effect of these factors is termed the ‘effective labeling efficiency’ (ELE). Although we are able to fully resolve labeled tRNA from unlabeled tRNAs by RP-HPLC, in practice our labeled preparations typically contained 0.8–0.9 dye/tRNA. However, because unlabeled cognate tRNA, typically outcompetes the labeled cognate tRNA for binding to the ribosome 2- to 5-fold, the presence of even 10–20% of unlabeled tRNA in our preparations leads to ELEs of 40–50%. Thus, labeled tRNAs account for only 40–50% of cognate tRNA binding events, limiting the length of criterion events we could choose, the complexity of mRNA mixtures and the identification criteria we could apply successfully. In L1-tRNA experiments, the ELE results in missed events when a single unlabeled tRNA accommodates into the ribosome. In the tRNA-tRNA method, on the other hand, both adjacent tRNAs must be labeled to produce FRET, so the proportion of detected events is reduced by the product of the two ELEs. In addition, using our current TIRF approach, background from unbound tRNAs restricts concentrations of directly excited labeled tRNA to values (<50 nM) much below those found physiologically (>µM), consequently causing a slowdown in translation rate. Higher labeled tRNA concentrations can be employed in L1-tRNA because the acceptor-labeled tRNAs are only excited by energy transfer from a nearby fixed donor on the ribosome.

Both of these advantages for L1-tRNA may become less important in future experiments. The availability of labeled tRNAs with higher ELEs that contain very little unlabeled tRNA (<1–2%) and/or compete more effectively against unlabeled tRNA would increase mRNA identification efficiency and accuracy. Korlach et al [23] overcame similar problems in producing fluorescent-labeled deoxynucleotide triphosphate derivatives for use in sequencing DNA. Strategies under consideration include the use of mutant strains that would result in tRNAs containing a unique DHU position that can be labeled [16] as opposed to wild-type tRNAs, for which labeling is generally distributed over two or more DHU positions [24], [25]; and the introduction of fluorophores at other than DHU positions [22], [26], [27], [28]. Further improvements that would improve monitoring of more complex mixtures of mRNAs include decreasing the rates of dye photobleaching and of software algorithmic misidentification due to noise in the fluorescence intensity traces.

Porting the concept of peptide identification from ribosome-derived fluorescent signals into live cells [20] would be a major complement to mass spectrometry and DNA microarrays for measuring gene expression during development, pathophysiological studies and pharmaceutical and peptide expression screens. The L1-tRNA FRET approach has more promise for monitoring protein synthesis in live cells than tRNA-tRNA FRET because it tracks individual ribosomes continuously. With sparse labeling of endogenous ribosomes in cells, for example via genetic modification linking a green fluorescent protein variant to ribosomal protein L1 [29], FRET signals could be collected from a fixed subset of translating complexes by following the donor fluorescence. This idea would not be feasible using tRNA-tRNA approach because adjacent ribosomes would randomly give rise to FRET signals making their timing and order unrelated to the sequential synthesis of any one peptide.

## Methods

### Ribosome preparation

70S, 50S lacking L1 protein (50SΔL1), and 30S ribosomes were prepared according to published procedures [30], [31], [32], [33]. Initiation complexes for tRNA-tRNA experiments were formed by mixture of 70S ribosomes with mRNA, initiation factors, and fMet-tRNAf^Met^ in buffer, and purified by centrifugation through a sucrose cushion [12]. In the presence of initiation factors, there is an active exchange between 70S ribosomes and 30S and 50S subunits. As a result, this method of forming 70SICs preserves the standard assembly mechanism while ensuring a 1∶1 subunit stoichiometry. For L1-tRNA experiments, the non-conserved amino acid position T202 of L1 was mutated to cysteine (T202C-L1) using the QuikChange Mutagenesis System (Stratagene, Inc.) and verified by DNA sequencing. The T202C-L1 was overexpressed for 18 hours at 20°C, and purified using TALON metal affinity resin (Clontech). The purified T202C-L1 was labeled using a maleimide-conjugated Cy3 dye (GE Biosciences), and separated from free dye on a G25 size exclusion column. The T202C-L1 (Cy3) was reconstituted into 50SΔL1 subunits, and reconstitution was analyzed by SDS-PAGE and [^35^S]-fMet-tRNA^fMet^ binding. The labeled 50S subunit was combined with purified 30S subunits in order to create 70S ribosomes prior to mixture with mRNA, initiation factors and fMet-tRNAf^Met^ to prepare initiation complexes as described [30], [31], [32], [33] for tRNA-tRNA experiments.

### Charged and Labeled tRNA preparation

Amino acid specific tRNAs, *E. coli* tRNA^fMet^, *E. coli* tRNA^Val^ yeast tRNA^Phe^, *E. coli* tRNA^Phe^, *E. coli* tRNA^Glu^ and *E. coli* tRNA^Tyr^ were purchased from Chemical Block, Inc. (Moscow) and prepared using the reduction, charging and labeling protocol as described [17]. Neither the charging nor the labeling reactions went to completion. Separations of charged from uncharged tRNAs were achieved by reversed-phase HPLC using a LiChrospher WP-300 RP-18 (5 µm bead) column (250–4 mm, Merck KGaA-Darmstadt). The tRNA mixture was applied to the column equilibrated with buffer A (20 mM NH_4_Ac pH 5.0, 10 mM MgAc_2_ and 400 mM NaCl) and the aminoacylated tRNAs were eluted with 20–30% buffer B (20 mM NH_4_Ac pH 5.0, 10 mM MgAc_2_, 400 mM NaCl and 30% [v/v] ethanol). Cy3/Cy5/Cy5.5 labeled tRNAs were separated from unlabeled tRNAs using the same column. Labeled tRNAs elute in 85–95% buffer B (pH 6.5). Stoichiometries of fluorophore/tRNA labeling varied from 0.7–1.2 probe per tRNA. The probes are equally distributed among the dihyrouridines in the “D” loop of these tRNAs [24].

### mRNA preparation

mRNAs - 1, -2, -5, and -6 were prepared via *in vitro* transcription and 3′-biotinylation, as described below. mRNAs -3 and -4 were purchased as 5′-biotinylated derivatives (Dharmacon RNAi Tech.) and used as received.

DNA fragments corresponding to mRNAs-1 and -2, were cloned into a pTZ18R vector, which contains a T7 promoter, through 3 steps by the SLIM PCR method [34]. The sequence in the DNA coding region was confirmed by sequencing. The DNA construct was linearized with Hind III and used as a template for transcription using the AmpliScribe T7-Flash *in vitro* Transcription Kit (Epicentre). The transcript was purified via phenol and chloroform extraction, followed by precipitation with 5 M LiCl and 95% Ethanol. The final RNA sample was dissolved in DEPC treated H_2_O. The integrity and purity of the mRNA was confirmed using agarose gel electrophoresis. For additional confirmation that this procedure generated the desired mRNA sequence, the transcript was reverse transcribed into cDNA using the 1^st^ Strand cDNA Synthesis Kit (Roche Diagnostics). The cDNA strand was purified via phenol and chloroform extraction followed by ethanol precipitation, and was subjected to DNA sequencing, confirming the correct sequence. mRNAs -5 and -6 were transcribed from DNA sequences obtained commercially (GenScript, Piscatawaty NJ) and inserted into pUC18 plasmids under the control of a T7 promoter sequence. The coding regions in the DNA plasmids were confirmed by DNA sequencing, linearized with HindIII treatment, transcribed into mRNA, and recovered as described above. The size and purity of the RNA transcripts were confirmed by agarose, and denaturing PAGE gel electrophoreses.

The mRNA biotinylation procedure was elaborated, with several modifications, from a previous procedure [35], which is based on selective periodate oxidation of RNA at its 3′ end and reaction of the oxidized product with biotin hydrazide. Typically, the oxidation of mRNA was performed in a solution containing mRNA at a concentration of 10–50 A_260_/ml, 100 mM sodium acetate (pH 5.0) and 90 mM sodium m-periodate (prepared fresh). After an incubation of 2 hours at room temperature, periodate was precipitated by adding KCl to a final concentration of 200 mM and incubating for 5 minutes on ice. The precipitate was removed by centrifugation for 5 minutes at 10000 g, 2°C and passage of the supernatant through a Sephadex G-25 column (Nap-5, Pharmacia). Biotin hydrazide (21339, Pierce) was then added to a final concentration of 2 mM from a 50 mM stock in DMSO (prepared fresh). The biotinylation reaction was carried out for 2 hours at room temperature, after which the whole mixture went through a Sephadex G-25 column (PD-10, Pharmacia). The biotinylated mRNA was obtained after perform ethanol precipitation on the eluted solution from column and dissolved in DEPC treated H_2_O. The concentration of biotinylated mRNA was determined by A_260_.

### Microscopy

Single molecule spectroscopic microscopy was performed on a home-built objective-type TIRF microscope based on a Nikon Eclipse Ti with an EMCCD camera (Cascade 512b) for sensitive photon detection and solid state lasers for excitation (532 nm and 640 nm) as described before [12]. For tRNA-tRNA experiments, alternating excitation (ALEX) [36] between 532 nm and 640 nm every frame was achieved with an acousto-optic tunable filter (AOTF, AA Opto-Electronic, Inc.) synchronized with the camera. Fluorophore emission from the fluorophores collected by the microscope was spectrally separated by interference dichroic and interference bandpass filters. For tRNA-tRNA FRET, a Dual-View or Quad-View imaging system (Photometrics, Inc., Tucson, AZ) was used for spectral separation of emission from the two fluorophores, resulting in 46 µm×92 µm (256×512 pixels) or 46 µm×46 µm (256×256 pixels) recording fields. The dichroic mirror had transition wavelength at 630 nm and the bandpass filters were 585/70 nm and 680/50 nm. For L1-tRNA FRET, in order to facilitate experimentation with different dyes, a similar home-built imaging system was used to separate emission from the donor and two acceptor dyes. For Cy3, Cy5 and Cy5.5, dichroic filters with 625 nm and 680 nm transition wavelengths and 570/60 nm, 685/70 nm and 710/50 nm bandpass filters were used.

Initiation complexes (ICs) were specifically attached via biotinylated message to PEG-passivated slides decorated with biotin-PEG to which streptavidin was attached. For both tRNA-tRNA and L1-tRNA experiments, 1 nM IC was usually incubated in the slide for 3 min and then washed out with buffer. Data collection started 10 seconds prior to injection of the translation mixture, which contained 10 nM preformed labeled TC, 100 nM preformed unlabeled TC, 2 µM EF-G, and 2 mM GTP, and lasted for 10 min. For tRNA-tRNA, a typical movie produced approximately 500 FRET traces. For L1-tRNA experiments, approximately 100 FRET traces were analyzed per field for mRNAs 3 and 4, and due to photobleaching of the Cy3 on L1, fewer (10–20) FRET traces for the other (longer) mRNAs. For experiments with mixtures of initiation complexes, the separately purified initiation complex samples were mixed in various proportions (as indicated in Figs. 4 and S4) before adding the mixture to the sample chamber. Unless specifically mentioned, experiments were performed in TAM_15_ buffer (15 mM MgAc_2_, 50 mM Tris-HCl pH 7.5, 30 mM NH_4_Cl, 70 mM KCl, and 1 mM DTT) with an oxygen scavenging system to prolong and stabilize the fluorophores (3 mg/mL glucose, 100 µg/mL glucose oxidase (Sigma-Aldrich), 40 µg/mL catalase (Roche), and 1.5 mM 6-hydroxy-2,5,7,8-tetramethyl-chromane-2-carboxylic acid (Trolox, Sigma-Aldrich – by dilution from a DMSO stock solution)). In tRNA-tRNA experiments, the photobleaching is almost negligible [13], Cy3 and Cy5 on tRNA averaging ∼1000 seconds before photobleaching. The contribution of photobleaching to disappearance of Cy3 and Cy5 is about 0.6–1.6%. For L1-tRNA FRET, donor photobleaching averaged ∼20 s leading to decreased readable traces per recording field for longer mRNAs, as mentioned.

To test tRNA binding to the E-site of the ribosome, a buffer containing polyamines (4.5 mM MgAc2, 2 mM spermidine, 0.05 mM spermine, 20 mM Hepes-KOH pH 7.3, 150 mM NH4Ac, 4 mM β-mercaptoethanol) was used in Fig. S3.

## Supporting Information

Figure S1 **Multiple criterion events detected by tRNA-tRNA FRET between Cy3-F and Cy5-V during translation of mRNA-3 (A), mRNA-4 (B), and mRNA-5 (C).** Color coding as described in Fig. 1.
(TIF)

Figure S2 **Multiple criterion events detected by FRET between L1^Cy3^ and either Cy5.5-F or Cy5-V during translation of mRNA-3 (A), mRNA-4 (B), and mRNA-5 (C).** Color coding as described in Fig. 2.
(TIF)

Figure S3 **Binding of tRNA to the E-site.** (A) Non-specific E-site binding of Cy5.5-F in a buffer with polyamines (see Methods). Cy5.5-labeled deacylated Phe-tRNA was added to 70S initiation complex immobilized on the surface via biotin-labeled mRNA. Without EF-G, translation is halted, yet multiple binding events occur near the Cy3-labeled L1 protein. (B) A similar experiment, but in TAM15 buffer, which has no polyamines. In such experiments, very few FRET events are identified.
(TIF)

Figure S4 **Multiple criterion events detected by tRNA-tRNA FRET between Cy3-F and Cy5-V during translation of mRNA-6 (A). Analysis of mixtures of mRNA-2 and mRNA-6 (B).** Color coding as described in Fig. 4. The criterion for classifying mRNA-6 from mRNA-2 is the occurrence of both FV and VF e
(TIF)
