# Supplementary figures and images for: FRET-Based Identification of mRNAs Undergoing Translation
Source: PLoS One. 2012 May 31;7(5):e38344. doi: 10.1371/journal.pone.0038344 (PMC3365013; doi:10.1371/journal.pone.0038344)

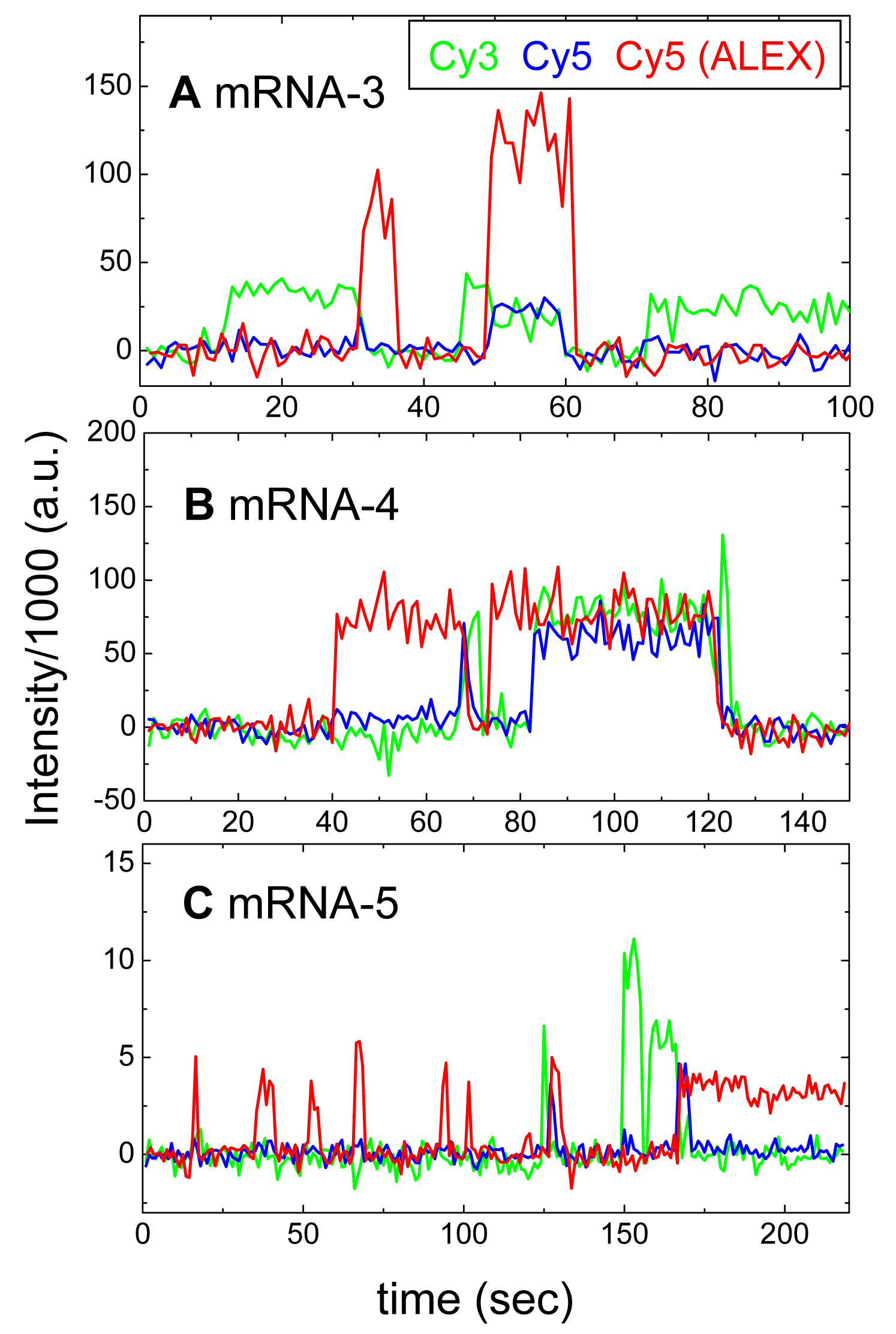

Supplement: Figure S1 — Multiple criterion events detected by tRNA-tRNA FRET between Cy3-F and Cy5-V during translation of mRNA-3 (A), mRNA-4 (B), and mRNA-5 (C). Color coding as described in Fig. 1. (TIF) [file pone.0038344.s001.tif]

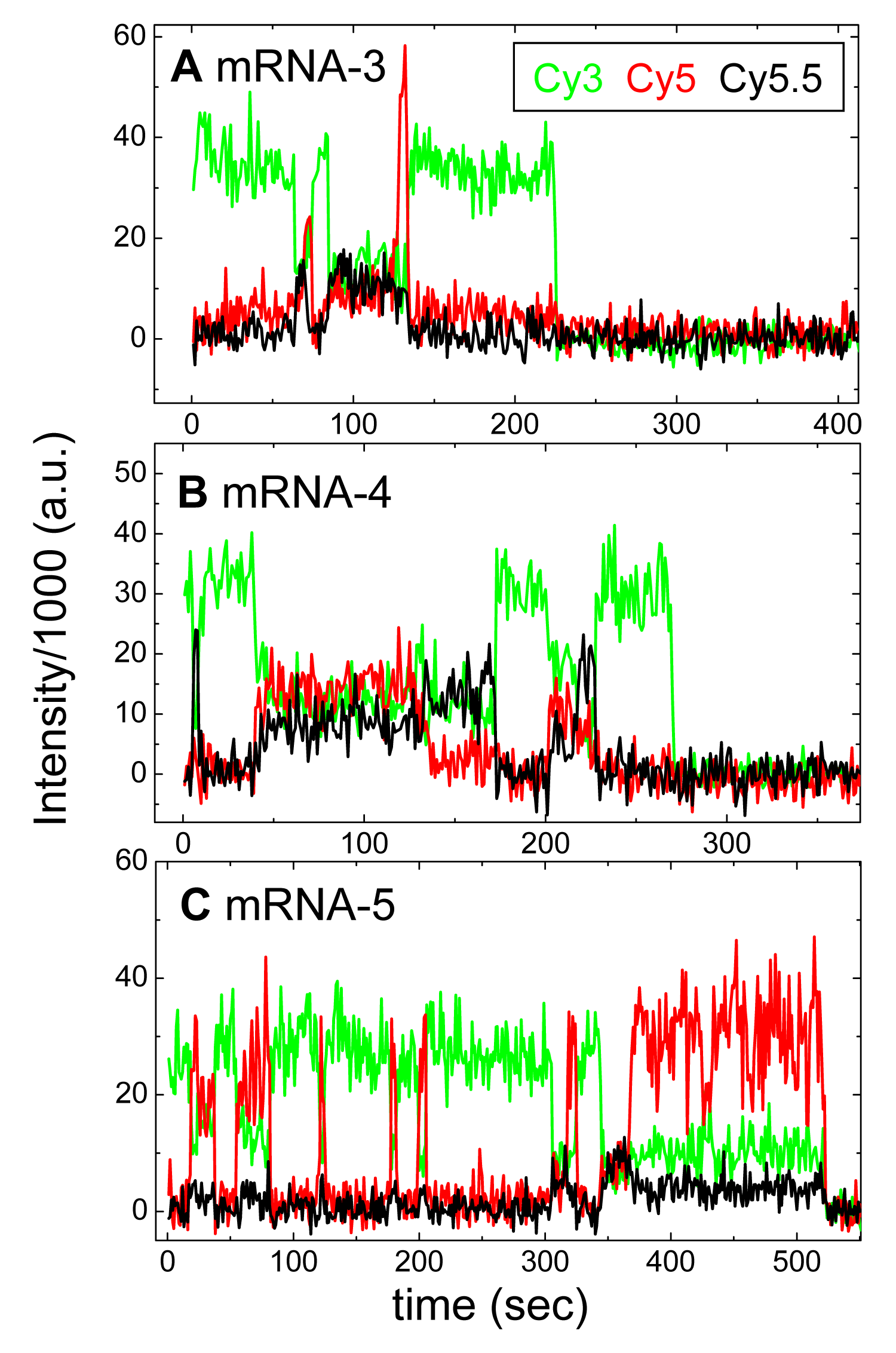

Supplement: Figure S2 — Multiple criterion events detected by FRET between L1Cy3 and either Cy5.5-F or Cy5-V during translation of mRNA-3 (A), mRNA-4 (B), and mRNA-5 (C). Color coding as described in Fig. 2. (TIF) [file pone.0038344.s002.tif]

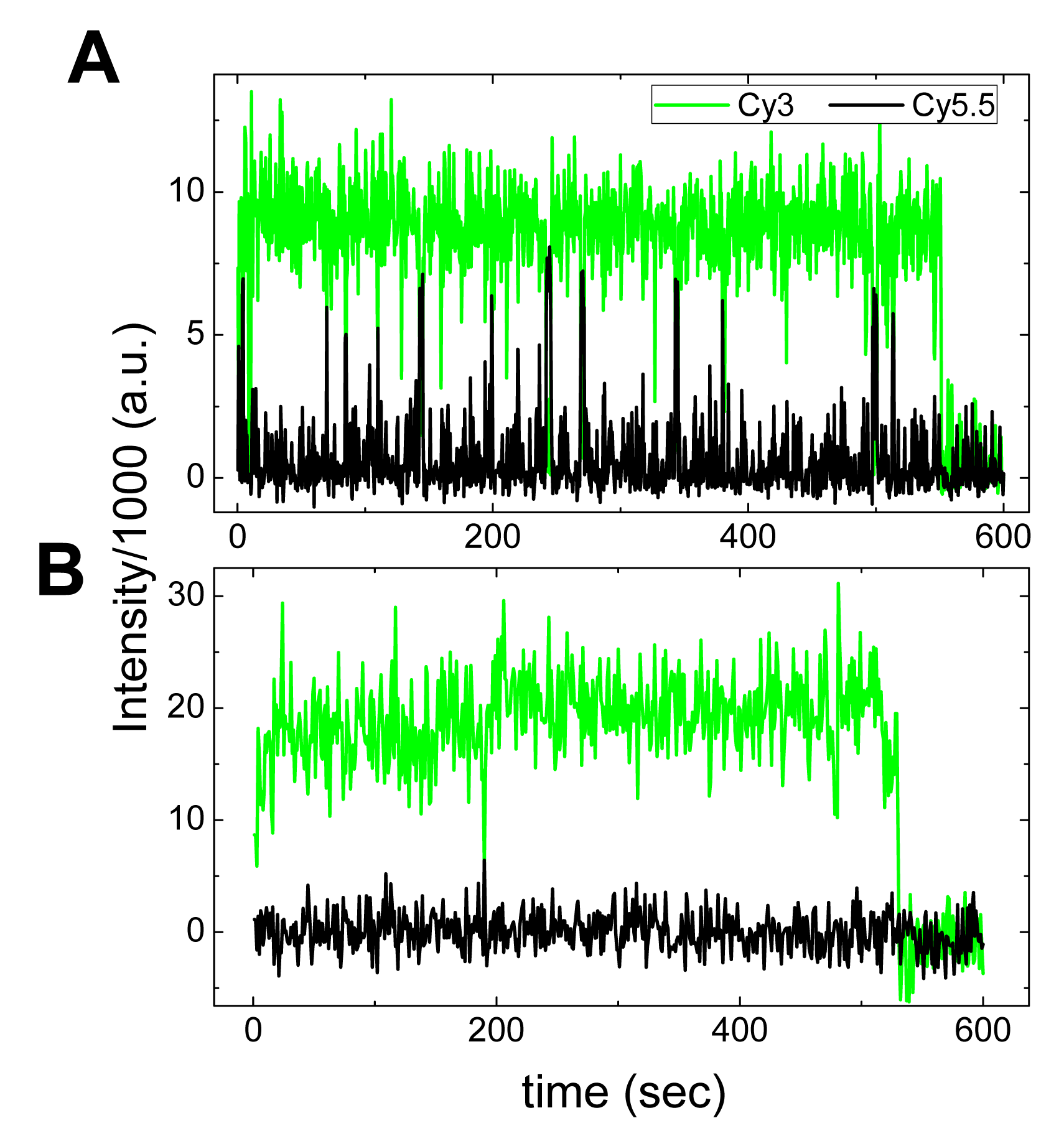

Supplement: Figure S3 — Binding of tRNA to the E-site. (A) Non-specific E-site binding of Cy5.5-F in a buffer with polyamines (see Methods). Cy5.5-labeled deacylated Phe-tRNA was added to 70S initiation complex immobilized on the surface via biotin-labeled mRNA. Without EF-G, translation is halted, yet multiple binding events occur near the Cy3-labeled L1 protein. (B) A similar experiment, but in TAM15 buffer, which has no polyamines. In such experiments, very few FRET events are identified. (TIF) [file pone.0038344.s003.tif]

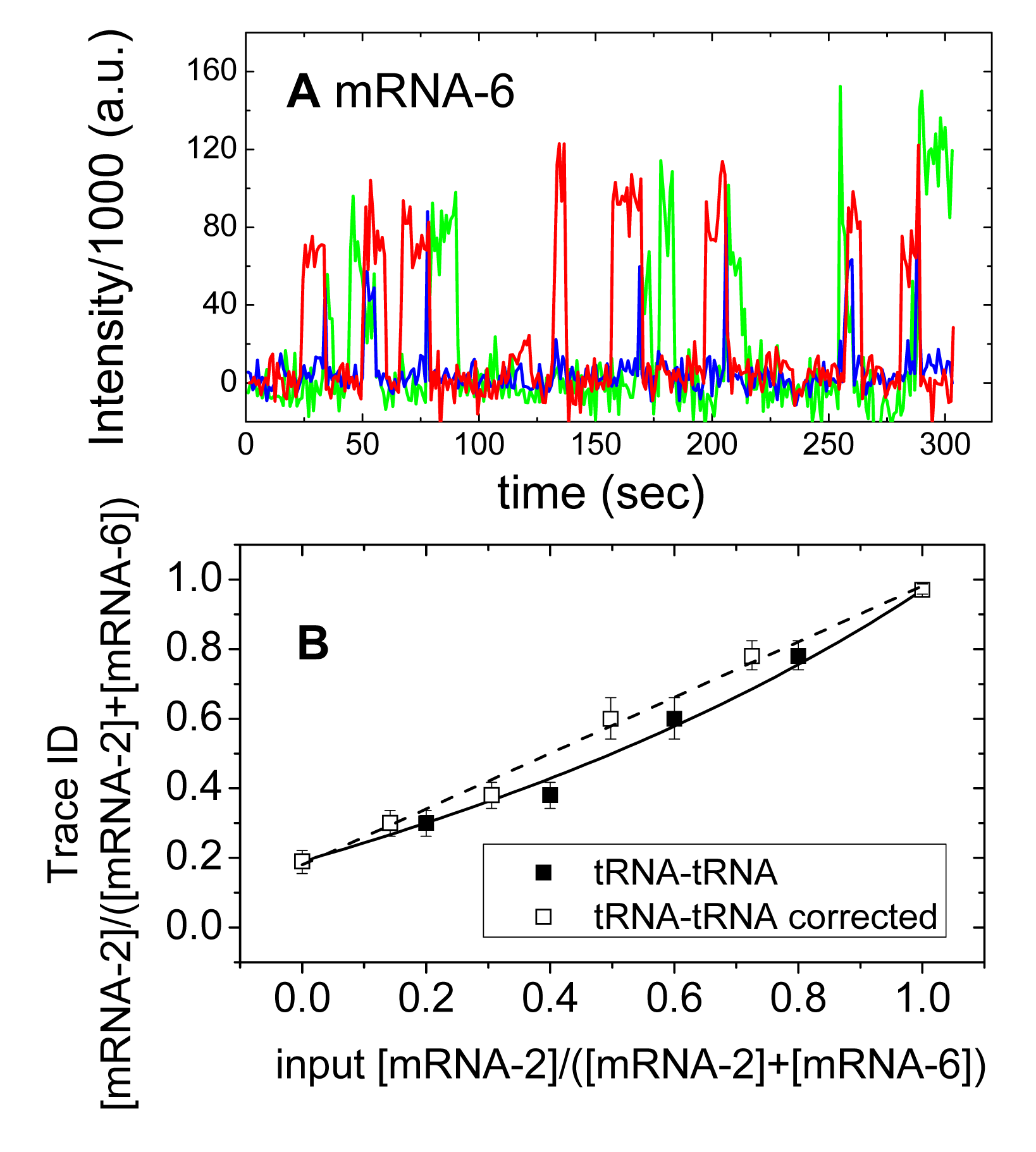

Supplement: Figure S4 — Multiple criterion events detected by tRNA-tRNA FRET between Cy3-F and Cy5-V during translation of mRNA-6 (A). Analysis of mixtures of mRNA-2 and mRNA-6 (B). Color coding as described in Fig. 4. The criterion for classifying mRNA-6 from mRNA-2 is the occurrence of both FV and VF e (TIF) [file pone.0038344.s004.tif]
